# Prognostic Value of an Immune Long Non-Coding RNA Signature in Liver Hepatocellular Carcinoma

**DOI:** 10.4014/jmb.2308.08022

**Published:** 2024-01-30

**Authors:** Rui Kong, Nan Wang, Chun li Zhou, Jie Lu

**Affiliations:** 1 Department of Gastroenterology, Suzhou Municipal Hospital, The Affiliated Suzhou Hospital of Nanjing Medical University, Gusu School, Nanjing Medical University, P.R. China; 2 Department of Gastroenterology, Pu Dong Area Gongli Hospital, School of Medicine, Shanghai University, Shanghai 200135, P.R. China; 3 Department of Gastroenterology, Shanghai General Hospital, Shanghai Jiao Tong University School of Medicine, Shanghai 200080, P.R. China

**Keywords:** Long non-coding RNA, immune prognostic signature, hepatocellular carcinoma, overall survival

## Abstract

In recent years, there has been a growing recognition of the important role that long non-coding RNAs (lncRNAs) play in the immunological process of hepatocellular carcinoma (LIHC). An increasing number of studies have shown that certain lncRNAs hold great potential as viable options for diagnosis and treatment in clinical practice. The primary objective of our investigation was to devise an immune lncRNA profile to explore the significance of immune-associated lncRNAs in the accurate diagnosis and prognosis of LIHC. Gene expression profiles of LIHC samples obtained from TCGA database were screened for immune-related genes. The optimal immune-related lncRNA signature was built via correlational analysis, univariate and multivariate Cox analysis. Then, the Kaplan-Meier plot, ROC curve, clinical analysis, gene set enrichment analysis, and principal component analysis were performed to evaluate the capability of the immune lncRNA signature as a prognostic indicator. Six long non-coding RNAs were identified via correlation analysis and Cox regression analysis considering their interactions with immune genes. Subsequently, tumor samples were categorized into two distinct risk groups based on different clinical outcomes. Stratification analysis indicated that the prognostic ability of this signature acted as an independent factor. The Kaplan-Meier method was employed to conduct survival analysis, results showed a significant difference between the two risk groups. The predictive performance of this signature was validated by principal component analysis (PCA). Additionally, data obtained from gene set enrichment analysis (GSEA) revealed several potential biological processes in which these biomarkers may be involved. To summarize, this study demonstrated that this six-lncRNA signature could be identified as a potential factor that can independently predict the prognosis of LIHC patients.

## Introduction

Liver hepatocellular carcinoma, a prevalent malignancy globally, exhibits escalating rates of mortality and incidence [1, 2]. The primary approach utilized in LIHC administration is surgery, however, many patients are in the middle-advanced stage at first diagnosis and miss the chance of accepting surgery [3-5]. In a broad sense, liver is classified as a lymphoid organ [6]. It has been documented that during tumor progression, immunological tolerance is influenced by various factors including cytokines, hepatic nonparenchymal cells, dendritic cells, and lymphocytes, which actively modulate this process [7-10]. Meanwhile, immunology therapy comprising immune checkpoints, adoptive cellular immunotherapy (ACT) and vaccines has presented promising possibilities for the treatment of liver cancer (LIHC). These advancements have significantly broadened the horizons of LIHC treatment [11, 12]. Several studies have clarified that clinical application of immune checkpoint blockade programmed cell death-1(PD-1) and cytotoxic T-lymphocyte antigen-4 (CTLA4) has enhanced the survival rate of some advanced patients [13-15]. Consequently, there is an urgent requirement for the study of immune biomarkers that exhibit both high sensitivity and specificity in terms of diagnosing and predicting the prognosis of hepatocellular carcinoma (LIHC). Long noncoding RNA is a type of poorly conserved RNA in length from 200 base pairs to 100 kilobase pairs. This particular RNA is capable of modulating gene expression at four primary levels: epigenetic regulation, epigenetic transcriptional regulation, posttranscriptional regulation and translational regulation [16-21]. According to their location with respect to protein-coding mRNAs, lncRNAs can be categorized into four categories: antisense, pseudogene, long intergenic ncRNA and intronic lncRNA [22]. Recent research has brought to light the crucial significance of lncRNAs in the innate immune response and the development, differentiation, and activation of T cells [23, 24]. Additionally, certain investigations have examined the correlation between aberrant expression of lncRNAs and tumorigenesis, metastasis, diagnosis or prognosis [25-27]. For example, HULC, a lncRNA that is specifically situated on cell plasma, exhibits a significant expression in hepatoma cells and promotes cell proliferation [28, 29]. In HBV-associated HCC, H19 has been documented to exhibit upregulated levels and represses the metastasis of tumors [30]. Through bioinformatic analysis, we developed a reliable immunological lncRNA model which serves as a valuable tool for facilitating the diagnosis and prognosis of liver hepatocellular carcinoma (LIHC). The long non-coding RNAs AC009005.1, AC099850.3, AL031985.3, AL117336.3, AL365203.2 and MSC−AS1 were critical components of the whole model. The data suggested that a high expression level of these biomarkers was positively correlated with poor survival and malignant phenotypes in the TCGA dataset. Univariate Cox regression and multivariate Cox regression analyses further clarified that this signature had an independent influence on overall survival. The results derived from KM plot, ROC curve, and PCA further proved the sensitivity and reliability of this prognostic model.

## Methods

### Data Source and Processing

RNA-sequencing data of LIHC samples were retrieved from the TCGA repository [31]. The data was generated using the Illumina HiSeq RNA-Seq platform. Additionally, we collected the corresponding clinical data, including survival time, TNM classification information, and risk factors. The dataset consisted of a total of 424 samples, with 50 being normal and 374 being primary hepatocellular carcinoma. The utilization and acquisition of this data were conducted in accordance with TCGA data access policies and publication guidelines. In our study, we excluded clinical samples that did not have precise outcomes or had follow-up times of less than 30 days. To match the names of mRNAs and long non-coding RNAs in the ensemble, we utilized the human general transfer format provided by the ensemble website [32].

### Selection of Immune-Related Long Non-Coding RNAs

**Construction of the Prognostic LncRNA Signature and Statistical Analysis**

The gene sets for 'immune responsé and 'immune system process' were obtained from the Molecular Signatures Database [33]. These gene sets were then used to identify immune-related genes in LIHC samples. Correlation analysis was performed on these genes using the 'limma' packages and the 'cor function' in R. The filter criteria were set as absolute cor (corresponding coefficients) > 0.4 and adjusted *P* value < 0.001 to identify the correlated lncRNAs. The network was visually represented using the Cytoscape software [34]. To ensure data reliability, specific criteria were applied to filter out lncRNAs that did not meet the following conditions: (1) lncRNAs with expression levels (FPKM ≥ 1) in at least 50% of the samples from patients with LIHC; and (2) lncRNAs that demonstrated consistent expression levels across all samples with no significant fluctuations [35]. We conducted a Univariate Cox regression analysis to identify a subset of candidate lncRNAs that exhibited a significant correlation (*P* < 0.001) with patient overall survival (OS). Subsequently, we performed multivariate Cox regression analysis to develop a prognostic lncRNA model. To validate the predictive significance of the 5-lncRNA signature in patients with HCC, we calculated risk scores for the test group of patients. These scores were determined using a formulated equation that incorporates the expression levels of the 6 lncRNAs. The formulas for calculating the risk scores are provided below. The tumor samples were categorized into a high-risk cohort and a low-risk cohort by applying the risk score threshold. To determine the survival rate, we performed Kaplan-Meier survival analysis. Additionally, we evaluated the performance of this long non-coding RNA (lncRNA) model using receiver operating characteristic (ROC) curve analysis. The optimization process involved the use of the Akaike Information Criterion (AIC). For all these statistical analyses, we utilized the R platform and the following packages: 'survival,' 'survminer,' 'survival ROC,' and 'pheatmap'.

lncRNA risk score = |coef|* expression value

risk score of samples = ∑ (lncRNA risk score)

### Analysis of the Clinical Features of the LncRNA Signature

Following the preceding analysis, we obtained the expression matrix of lncRNA biomarkers in individuals diagnosed with LIHC. We subsequently merged the clinical information with the expression data. We then examined the relationship between the expression of lncRNA biomarkers (significance level: *P* < 0.05) and different clinical features, including tumor stage (T stage), histologic grade (G stage), and pathological stage (S stage). This analysis was performed using the 'ggpubr' package in the R programming language.

### Gene Set Enrichment Analysis

The utilization of Gene Set Enrichment Analysis (GSEA) allowed for the investigation of the underlying relationship between risk scores obtained from co-expression analysis. For reference purposes, two sets of immune genes, namely 'immune system process' (M13664 genes annotated by GO term GO:0002376) and 'immune responsé (M19817 genes annotated by GO term GO:0006955), were obtained from the Molecular Signatures Database (MSigDB). Enrichment outcomes were considered statistically significant if they met the criterion of FDR < 0.25.

### Principal Component Analysis

Principal component analysis (PCA) was conducted to visualize the separation of samples with different risk scores based on the six-lncRNA signature, immune lncRNAs, immune-related genes, and all genes. Prior to PCA, the expression matrices were preprocessed by deduplicating the values through averaging and excluding any data with no change in expression level. The resulting graphs exhibited the three major components (PC1, PC2, PC3) in a three-dimensional space. The analysis was performed using the 'limma' and 'scatterplot3d' packages.

### Statistics

Data expression was performed using the mean ± SD. Two-group comparisons were analyzed using the Student's *t*-test, while multigroup comparisons were analyzed using one-way ANOVA. Spearman's correlation analysis was used to evaluate expression correlation. Kaplan Meier analysis was conducted to analyze overall survival. A *P* value < 0.05 was considered statistically significant.

## Results

### Construction of the Six-LncRNA Signature

**Fig. 1:**
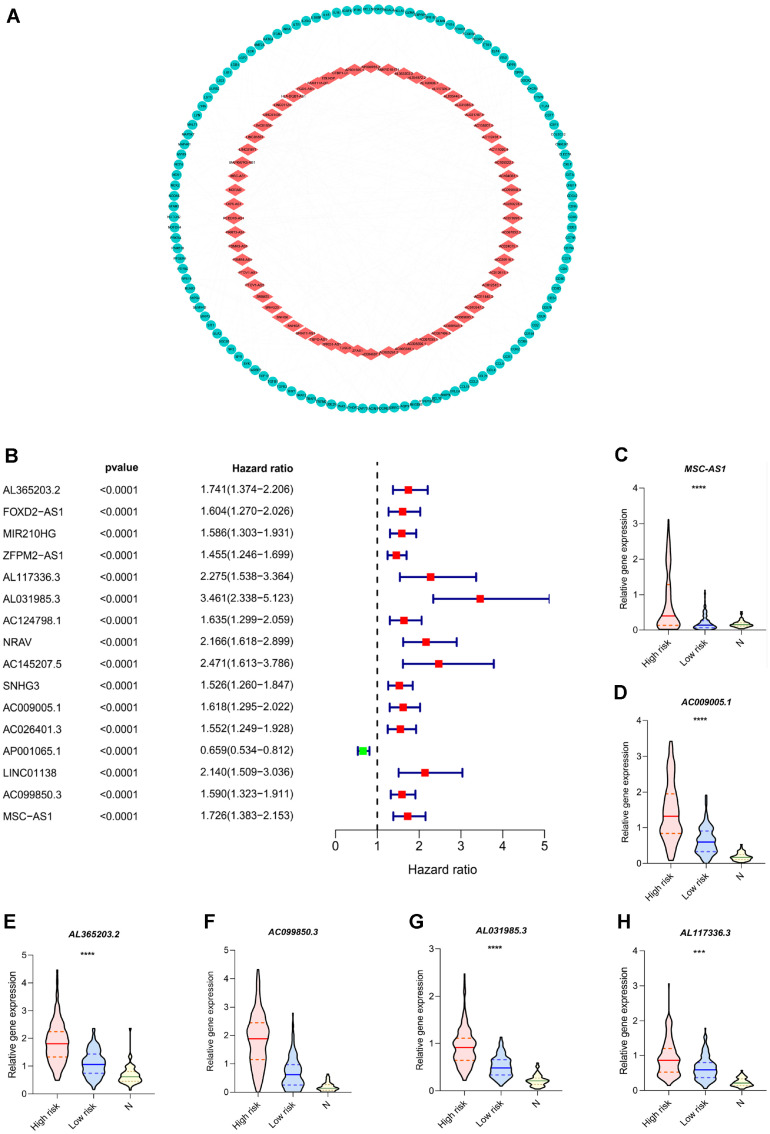
Construction of an immune lncRNA signature for liver hepatocellular carcinoma. (**A**) The network of partial immune genes and associated lncRNAs. **B**. Candidate lncRNA for the prognostic model with information about hazard ratio. (**C**) Relative gene expression of MSC-AS1 among the low-risk group, high-risk group, and non-tumor samples. (**D**) Relative gene expression of AC009005.1 among the low-risk group, high-risk group, and non-tumor samples. (**E**) Relative gene expression of AL365203.2 among the low-risk group, high-risk group, and non-tumor samples. (**F**) Relative gene expression of AC099850.3 among the low-risk group, high-risk group, and non-tumor samples. (**G**) Relative gene expression of AL031985.3 among the low-risk group, high-risk group, and non-tumor samples. (**H**) Relative gene expression of AL117336.3 among the low-risk group, high-risk group, and non-tumor samples.

In the present research, the transcriptome data of both LIHC tissues (*n* = 374) and normal tissues (*n* = 50) were obtained from the TCGA database. To classify the immune genes based on the patients' gene expression patterns, immune gene sets such as "immune response" and "immune system process" were utilized from the GSEA. Further correlational analysis was conducted on a total of 331 immune-related genes that were retained. Using R, lncRNAs meeting the criteria of |cor| > 0.4 and *P* < 0.001 were identified as potential candidates. Fig. 1A illustrates the visual representation of connections between immune genes and linked lncRNAs using Cytoscape software. Table 1 provides information on the coefficient and attributes of positive or negative regulation. Additionally, the candidate lncRNAs underwent both univariate and multivariate Cox regression analyses. Among them, 16 lncRNAs exhibited a strong association with survival outcomes (*P* < 0.0001). From these, 6 high-risk prognostic lncRNAs were identified (Fig. 1B). The prognostic optimization model yielded an AIC value of 1156.35. For detailed information on the six-lncRNA signature, please refer to Table 2. Based on the risk score, LIHC samples were divided into two groups: a high-risk group (*n* = 171) and a low-risk group (*n* = 172). Subsequently, the relative RNA expression levels of the six-lncRNA signature were assessed across different groups. Interestingly, the results indicated a significant upregulation of all six lncRNAs in LIHC tissues (Fig. 1C-1H).

### Prognostic Value of the LncRNA Signature for Assessing Clinical Outcome

**Fig. 2:**
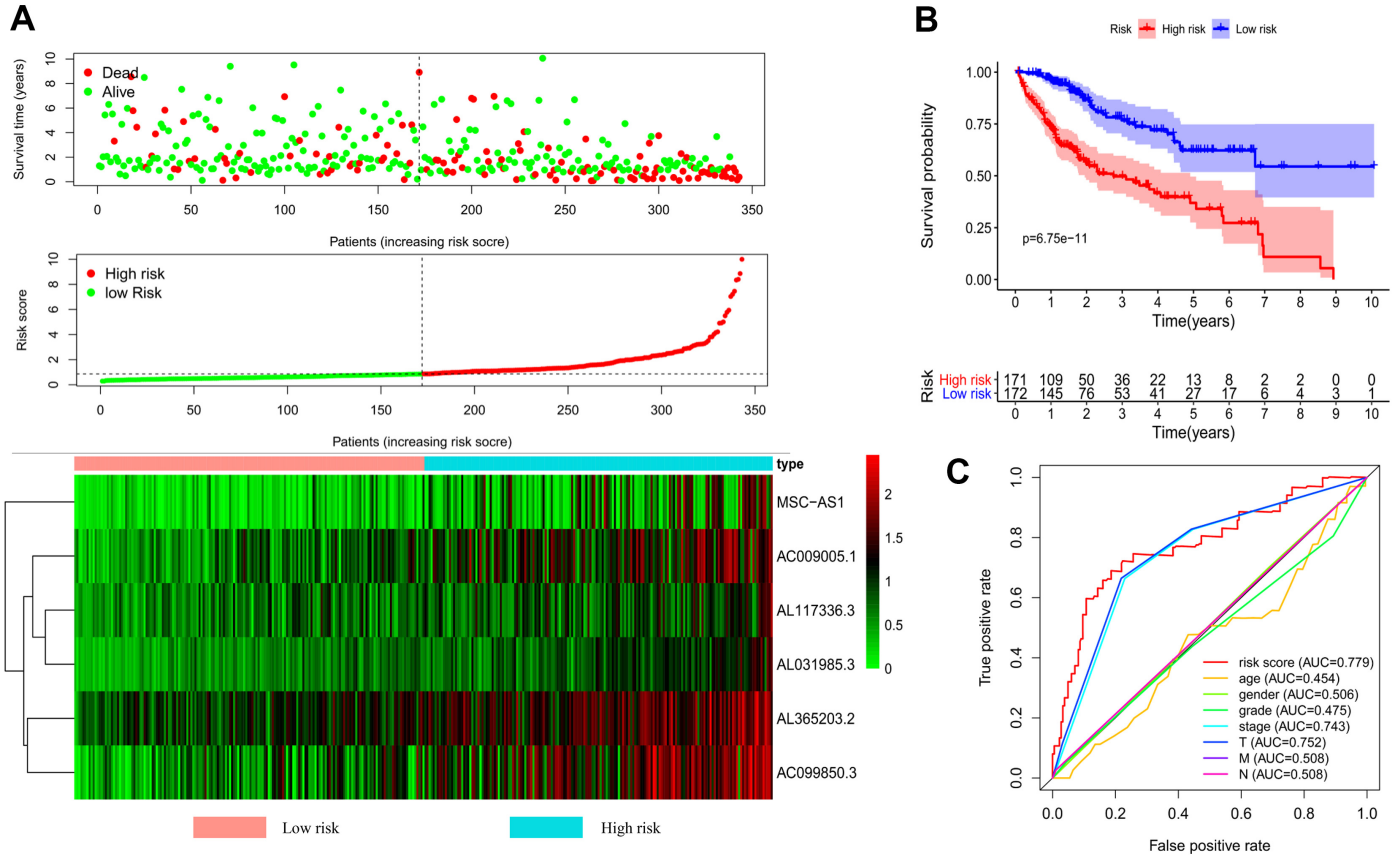
Validation of the prognostic lncRNA signature for hepatocellular carcinoma. (**A**) The upper graph shows the relationship between survival time and risk score, the medium graph shows the distribution of lncRNA risk score, the bottom heat map shows expression patterns of six-lncRNA signature for LIHC patients. (**B**) The Kaplan-Meier curve of different tumor groups based on the median risk score. (**C**) The ROC curve for the risk score, age, gender, grade, and TNM stage.

In Fig. 2A, we present the final survival state and expression profiles of the six-lncRNA signature for each sample, aiming to assess its potential in predicting the prognosis of LIHC patients. The scatter graph demonstrates a clear correlation between increasing risk score and worsened survival estimate. Furthermore, our survival analysis reveals significantly lower death rates in the low-risk group compared to the high-risk group (*P* = 6.75E−11). In fact, the five-year survival rate was 62.2% in the low-risk group and only 34.1% in the high-risk group (Fig. 2B). We further employed an ROC curve to evaluate the predictive accuracy of the combined six-lncRNA signature. Interestingly, the results indicate that this signature outperforms other clinical parameters, such as age, sex, grade, and TNM staging, in terms of prediction. The risk score system achieved an AUC of 0.779 (Fig. 2C).

### Correlation between LncRNA Signature and Clinical Characteristics

**Fig. 3:**
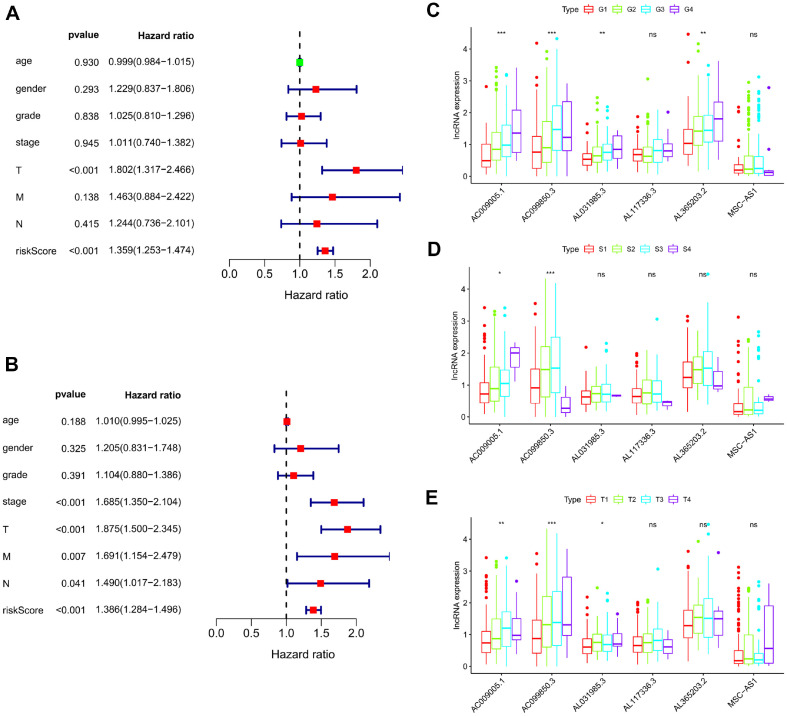
Evaluation of the six-lncRNA as an independent factor. (**A**) The tree diagram of multivariate analysis shows the statistical significance and hazard ratios of several indices as prognostic factors. (**B**) The tree diagram of univariate analysis shows the statistical significance and hazard ratios of several indices as prognostic factors. (**C**) Boxplot indicates the correlation of lncRNA biomarkers’ expression and tumor grade. (**D**) Boxplot indicates the correlation of lncRNA biomarkers expression and TNM stage. (**E**) Boxplot indicates the correlation of lncRNA biomarkers expression and T-staging.

To assess the correlation between patients' clinical indicators and outcomes based on their risk score, we conducted a stratified analysis on a total of 373 samples obtained from the TCGA cohort (Table 3). Moreover, a univariate analysis highlighted a significant association between TNM staging, as well as the six-lncRNA signature, with overall survival (OS). Furthermore, through multivariate analysis, T staging along with the six-lncRNA signature emerged as potential independent prognostic factors (Fig. 3A and 3B). Additionally, we investigated the relationship between the expression of the six lncRNAs and tumor stage as well as tumor grade. Our findings indicated a strong connection between the expression of AC009005.1, AC099850.3, AL031985.3, and MSC−AS1 with clinical grade. Additionally, AC009005.1 and AC099850.3 displayed associations with TNM staging, while AC009005.1, AC099850.3, and AL031985.3 levels were found to be correlated with T staging (Fig. 3C-3E).

### Gene Set Enrichment Analysis

**Fig. 4:**
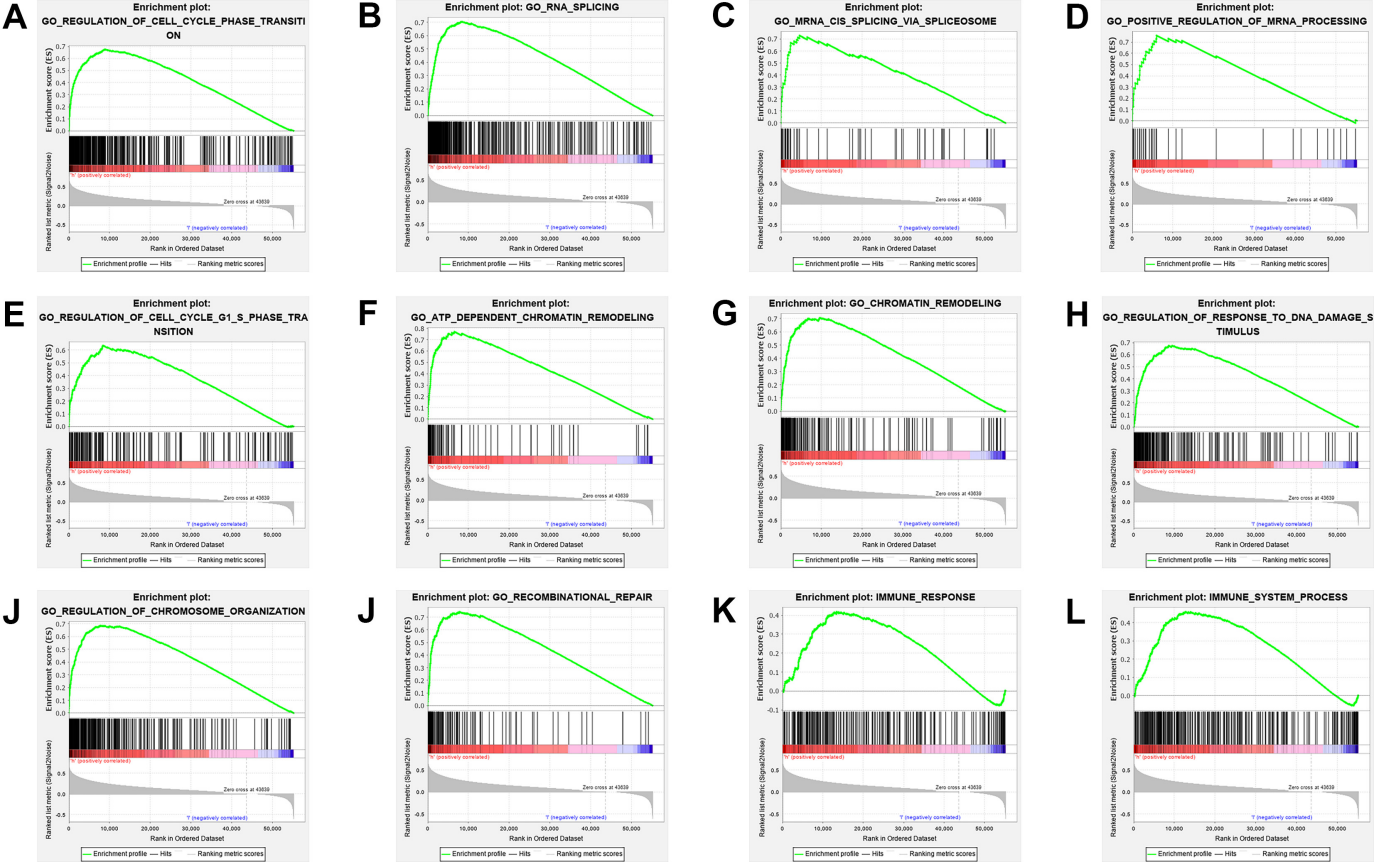
Highly enriched biological pathways for corresponding immune genes of six-lncRNA signature in TCGA. (**A**) Regulation of cell cycle phase transition (FDR < 0.007), (**B**) RNA splicing (FDR < 0.004), (**C**) mRNA CIS splicing via spliceosome (FDR < 0.002) (**D**) Positive regulation of mRNA processing (FDR < 0.002) (**E**) Regulation of cell cycle G1 S phase transition (FDR < 0.002) (**F**) ATP dependent chromatin remodeling (FDR < 0.002) (**G**) Chromatin remodeling (FDR < 0.002) (**H**) Regulation of response to DNA damage stimulus (FDR < 0.002) (**I**) Regulation of chromosome organization (FDR < 0.002) (**J**) Recombinational repair (FDR < 0.001) (**K**) Immune response (FDR < 0.203) (**L**) Immune system process (FDR < 0.087).

The utilization of GSEA was employed in order to investigate the potential connection between biomarkers and biological processes based on the risk score. As depicted in Fig. 4A-4B, GSEA data exhibited that protein-coding genes coexpressed within the high-risk group demonstrated significant enrichment in the reference gene sets "immune response" and "immune system process" (with FDR *q*-value < 0.25). Additionally, Fig. 4C-4L showcases the top ten biological processes that were highly enriched, indicating that the coexpressed genes associated with the six-lncRNA signature potentially participate in various cellular activities such as cell cycle phase transition, RNA splicing, regulation of chromosome organization, etc.

### Principal Component Analysis

**Fig. 5:**
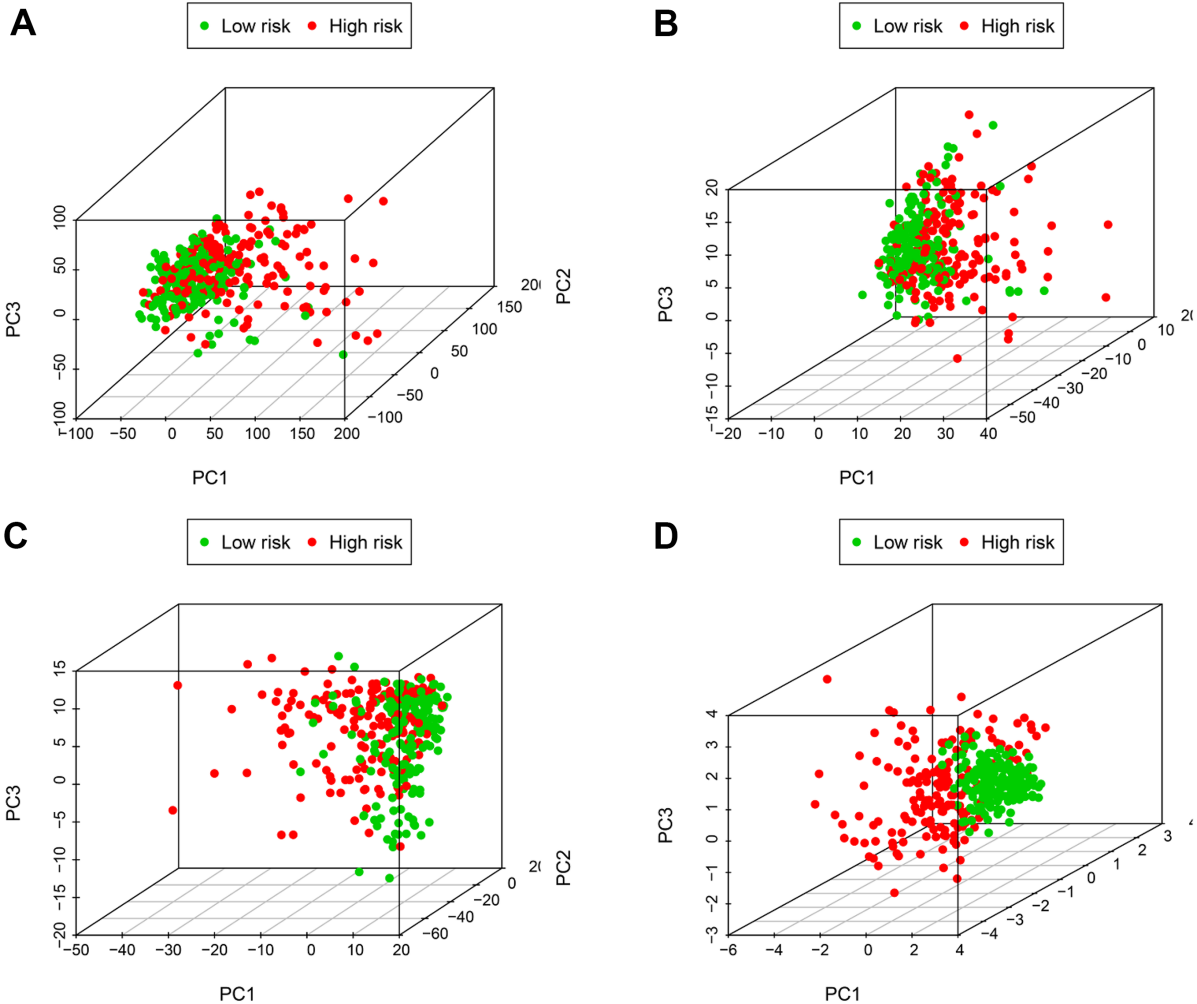
Principal component analysis of samples for TCGA. (**A**) PCA shows samples divisibility from the low risk- and high-risk group based on all gene expression. (**B**) PCA shows samples divisibility from the low risk- and high-risk group based on immune gene expression. (**C**) PCA shows samples divisibility from the low risk- and high-risk group based on immune lncRNA expression. (**D**) PCA shows samples divisibility from the low risk- and high-risk group based on lncRNA signature expression.

**Fig. 6:**
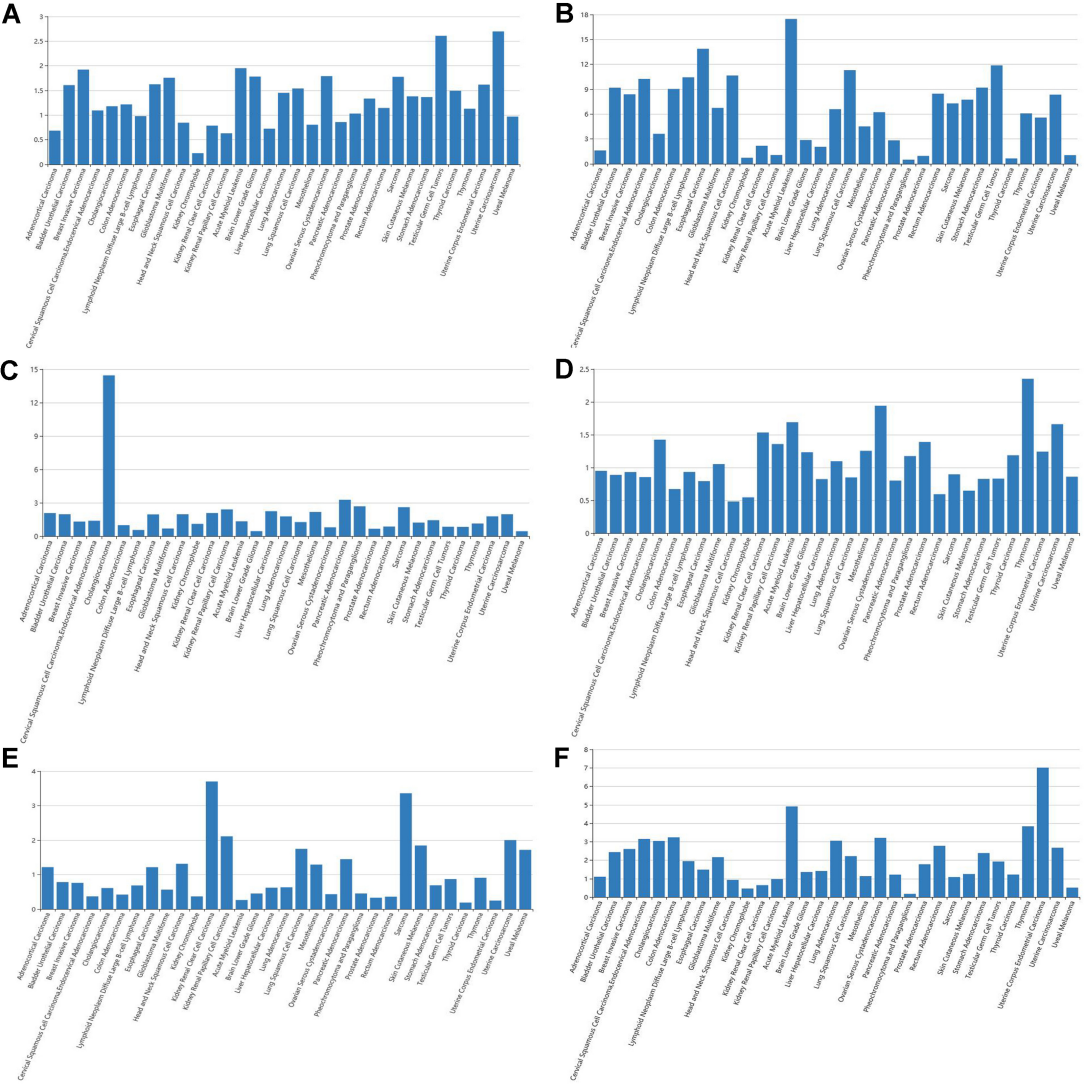
Expression profiles of the lncRNA signature in pan-cancer. (**A**) AL031985.3expression in pan-cancer. (**B**) AC099850.3 expression in pan-cancer. (**C**) AL365203.2 expression in pan-cancer. (**D**) AL117336.3 expression in pan-cancer. (**E**) MSC−AS1 expression in pan-cancer. (**F**) AC009005.1 expression in pan-cancer.

Principal component analysis (PCA) was conducted to aggregate samples based on gene expression patterns in two groups at risk. By reducing multiple indices and extracting main parameters, tumor samples were examined at four levels: lncRNA signature, immune lncRNAs, immune genes, and all genes. Sample data from the high-risk group is depicted by red points on the graph, while green points represent the low-risk group. Our analysis revealed that the six-lncRNA signature exhibited the highest degree of separation among the four levels (Fig. 5A-5D). These findings partially support the prognostic accuracy of the immune lncRNA model. Additionally, we assessed the expression profiles of these six lncRNAs in pan-cancers using the Lnc2Catlas database, and the resulting boxplots are displayed in Fig. 6.

## Discussion

Long noncoding RNA has drawn widespread attention as a potential molecular target for cancer diagnosis and treatment in recent years. Multiomics has developed rapidly following the application of high-throughput screening to cancer diagnosis and therapy [36-38]. Several studies have proposed that long noncoding RNAs are involved in tumor genesis and progression and that they contribute to the immune system [39-41]. For example, Jiang *et al*. demonstrated that a high level of lnc-EGFR in Tregs could enhance immunosuppression in hepatoma cells via activating AP-1/NF-AT1signaling [42]. Moreover, immunotherapy has advanced dramatically, the administration of anti-PD-1 antibodies has shown definite efficacy in virus-associated hepatocellular carcinoma [43, 44]. In this study, the potential role of immune lncRNAs as prognostic or therapeutic biomarkers was examined, and this finding may provide some insights for further research. From the TCGA dataset, we discovered a predictive immune pattern consisting of six lncRNAs: MSC−AS1, AC009005.1, AL117336.3, AL031985.3, AL365203.2, and AC099850.3. We subsequently confirmed its predictive significance for patients with HCC. The current investigation categorized patients into low-risk and high-risk groups according to the risk score of the previously mentioned lncRNAs. Univariate Cox analysis and multivariate Cox analysis were conducted to assess the suitability of these biomarkers. HCC was classified based on the TNM classification of the American Joint Committee on Cancer (AJCC) [45]. In addition, the AUC of the ROC system was higher than that of TNM classification and other indices, proving the validity of the proposed risk system as a predictor of overall survival independently. Several studies have indicated that MSC-AS1 is associated with the tumor microenvironment (TME) and can be utilized to evaluate the impact of immunotherapy and prognosticate hepatocellular carcinoma patients [46]. Moreover, the overexpressions of lncRNA AC009005.1 and AL365203.2 in HCC tumor tissue is correlated with an increased presence of a proinflammatory senescence-associated secretory phenotype (SASP), enhanced infiltration of regulatory T (Treg) cells, and reduced infiltration of naïve B cells [47]. Xing *et al*. demonstrated that patients with cervical squamous cell carcinoma and endocervical adenocarcinoma patients who exhibited down-regulated expression levels of lncRNAs AL117336 showed increased infiltration of immune cells and expression of various immune checkpoints. These findings suggest that these patients may derive greater benefits from immune checkpoint blockade therapy [48]. Additionally, lncRNA AC099850.3 was found to promote malignant behavior in HCC cells, and analysis of immune cell infiltration revealed a positively correlation between AC099850.3 and T follicular helper cells, M0 macrophages, CD4+ memory T cells, and memory B cells [49]. Alpha-fetoprotein (AFP) is extensively employed in clinical practice for the diagnosis of liver cancer and assessment of treatment effectiveness [50-52]. In a physically normal human body, AFP expression remains minimal due to its synthesis by the fetal liver, which is subsequently downregulated after birth [53]. Notably, significant alternations in AFP serological levels are observed in approximately half of liver malignancies [54]. A concentration exceeding 400 ng/ml may suggest the presence of liver cancer [55]. Nevertheless, elevated AFP concentrations have also been documented in various other conditions, including cerebellar ataxia and testicular cancer [56, 57]. In the current stratified analysis, samples with AFP levels higher than 20 ng/ml (pathological threshold) were roughly concentrated in the high-risk group. These results inspired us that the expression of this six-lncRNA signature can be used together with serum AFP as an indicator for early diagnosis, efficacy assessment, and prognostic evaluation. In recent years, significant efforts have been made to examine lncRNAs in tumor immunology. Extensive research has been conducted to uncover the mechanism by which lncRNAs regulate the immune response [58]. For instance, lncRNA NKILA plays a crucial role in boosting immune evasion by associating STAT1 and NF-κB signaling in various tumors [59, 60]. LncRNA ID2-AS1 inhibits HCC metastasis by activating the HDAC8/ID2 pathway [61]. In this study, six prognostic biomarkers showed high expression levels in tumor samples. Analysis using GSEA revealed the enrichment of corresponding genes in a range of GO biological process pathways, such as the cell cycle, RNA splicing, DNA repair, and chromosome regulation. However, further investigation is required to determine the specific roles of these lncRNAs in regulating biological pathways.

## Conclusion

We have successfully devised an immune model comprising of six lncRNAs to forecast the outcome of LIHC samples. Our study extensively examined the bioinformatics analysis to evaluate the functionality and efficacy of this lncRNA signature. The results derived from the observations suggest that this signature holds the potential to introduce a novel method for precise diagnosis and prognosis. Nevertheless, it is crucial to conduct clinical trials and functional tests in order to comprehend the underlying mechanism before considering its applicability in a clinical setting.

## Abbreviations

ACT: adoptive cellular immunotherapy, AFP: alpha-fetoprotein, AIC: Akaike information criterion, AJCC: American Joint Committee on Cancer, AUC: area under the curve, BMI: body mass index, CTLA4: cytotoxic T-lymphocyte antigen-4, FPKM: fragments per kilobase of exon model per million mapped fragments, GO: gene ontology, GSEA: gene set enrichment analysis, LIHC: liver hepatocellular carcinoma, lncRNA: long non-coding RNA, OS: overall survival, PD-1: programmed cell death-1, ROC: receiver operating characteristic, PCA: principal component analysis, TCGA: The Cancer Genome Atlas, TME: tumor microenvironment, Treg cells: regulatory T cells.

## Figures and Tables

**Table 1:** Correlation of immune genes and associated lncRNAs.

Immune Gene	lncRNA	Cor	*P* value	Regulation
KMT2A	AP001318.2	0.460	5.45E-21	Positive
NCOA6	AC012510.1	0.462	3.55E-21	Positive
TRAF2	SREBF2-AS1	0.451	3.66E-20	Positive
RPS19	AC132192.2	0.614	4.03E-40	Positive
TRAF2	AL035446.1	0.463	3.12E-21	Positive
HELLS	AL360181.2	0.455	1.55E-20	Positive
PRKRA	AP002884.1	0.416	4.67E-17	Positive
RPS19	AL109615.3	-0.401	7.64E-16	Negative
APOA2	FLJ42351	-0.460	6.18E-21	Negative
NCK2	MSC-AS1	0.408	2.10E-16	Positive
KMT2A	AC009005.1	0.483	2.82E-23	Positive
ITGB2	AL031985.3	0.679	7.50E-52	Positive
DPP4	AL117336.3	0.512	2.18E-26	Positive
HDAC7	AL365203.2	0.462	3.49E-21	Positive
CKLF	AC099850.3	0.431	2.26E-18	Positive

**Table 2:** Detailed information of the six-lncRNA signature.

Gene symbol	Gene_ID	Location	coef
MSC-AS1	ENSG00000235531.8	chr8: 71828167-72002405	0.3293
AC009005.1	ENSG00000267751.4	chr19: 567212-571745	0.3111
AL117336.3	ENSG00000271335.4	chr10: 35314552-35320998	0.3428
AL031985.3	ENSG00000260920.2	chr1: 40464319-40466767	0.4886
AL365203.2	ENSG00000273038.2	chr10:32887255-32889311	0.2210
AC099850.3	ENSG00000265415.1	chr17:59202677-59203829	0.1741

**Table 3:** Relationship between the risk score of the lncRNA signature for OS and clinical features.

	Low risk/high risk	Pearson χ^2^	*P*
Age		0.072	0.788
> = 55	119/116		
< 55	53/55		
Gender		3.565	0.059
Female	47/63		
Male	125/108		
TNM stage		8.404	0.004
I/II	130/108		
III/IV	30/53		
G		12.032	0.001
G1/G2	123/91		
G3/G4	47/77		
AFP (ng/ml)		5.917	0.015
> = 20	58/94		
< 20	64/57		
BMI		0.213	0.645
> =25	80/73		
< 25	81/82		
Race		0.268	0.605
White	83/86		
Asian	77/71		
